# Overcoming the High Error Rate of Composite DNA Letters‐Based Digital Storage through Soft‐Decision Decoding

**DOI:** 10.1002/advs.202402951

**Published:** 2024-06-14

**Authors:** Yaping Xu, Lulu Ding, Shigang Wu, Jue Ruan

**Affiliations:** ^1^ Shenzhen Branch, Guangdong Laboratory of Lingnan Modern Agriculture, Genome Analysis Laboratory of the Ministry of Agriculture and Rural Affairs Agricultural Genomics Institute at Shenzhen, Chinese Academy of Agricultural Sciences 7 Pengfei Street Dapeng New District Shenzhen 518120 P. R. China; ^2^ National Engineering Laboratory for Big Data System Computing Technology Shenzhen University Shenzhen 518060 P. R. China

**Keywords:** composite DNA letter, DNA digital storage (DDS), error‐correcting code (ECC), soft‐decision decoding

## Abstract

Composite DNA letters, by merging all four DNA nucleotides in specified ratios, offer a pathway to substantially increase the logical density of DNA digital storage (DDS) systems. However, these letters are susceptible to nucleotide errors and sampling bias, leading to a high letter error rate, which complicates precise data retrieval and augments reading expenses. To address this, Derrick‐cp is introduced as an innovative soft‐decision decoding algorithm tailored for DDS utilizing composite letters. Derrick‐cp capitalizes on the distinctive error sensitivities among letters to accurately predict and rectify letter errors, thus enhancing the error‐correcting performance of Reed‐Solomon codes beyond traditional hard‐decision decoding limits. Through comparative analyses in the existing dataset and simulated experiments, Derrick‐cp's superiority is validated, notably halving the sequencing depth requirement and slashing costs by up to 22% against conventional hard‐decision strategies. This advancement signals Derrick‐cp's significant role in elevating both the precision and cost‐efficiency of composite letter‐based DDS.

## Graphical abstract

This study devise Derrick‐cp, a cutting‐edge soft‐decision decoding algorithm, tailed for DNA Digital Storage (DDS) utilizing composite letters. It effectively tackles the high letter error rate from nucleotide errors and sampling bias, a barrier to the letters' application in DDS. Derrick‐cp utilizes the error sensitivities among letters to predict and correct letter errors, enhancing the error‐correcting performance and cost efficiency.

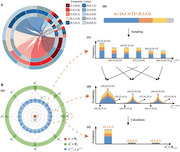

## 1 Introduction

In recent years, DNA digital storage (DDS) has emerged as a promising alternative to traditional electronic storage methods due to its exceptional density, longevity, and energy efficiency.^[^ ^1^ ^]^ Storing digital information on DNA involves encoding the information over the DNA alphabet (A, C, G, and T), synthesizing the sequences into DNA molecules for storage, and later retrieval. Reading the stored information requires sequencing the DNA oligos and decoding them back to the original form.

To convert digital information into DNA sequences, the common approach is directly mapping original files to four standard nucleotide letters (e.g., 00→A, 01→ C, 10→G and 11→T). At a given position in a sequence, there are four choices, leading to a theoretical upper limit of logical density of 2 bits per letter.^[^ ^2^ ^]^ Logical density herein refers to the amount of digital information that can be stored within a given unit length of DNA sequence, involved with storage capacity, and costs of DDS processes including synthesis, sequencing, and archiving. A recent study by Leon et al.^[^ ^3^ ^]^ proposed the use of composite DNA letters to substantially increase the logical density. Unlike traditional encoding approaches where each letter represents a single base represented by standard DNA nucleotide, a composite letter in this context consists of a group of four standard DNA nucleotides combined in a predetermined ratio. This unique design offers more than four choices per position, thereby achieving a logical density above the theoretical limit of 2 bits per letter. The in vitro experiments demonstrated a logical density of up to 4.29 bits per letter.^[^ ^3^ ^]^ Furthermore, using composite DNA has the potential to reduce the costs of DDS, due to the increased logical density leading to a decrease in DNA synthesis costs. Leon et al.^[^ ^3^ ^]^ has demonstrated that using current technologies, synthesis cost per position is approximately four orders of magnitude larger than sequencing cost per base, therefore a large composite alphabet could reduce the overall cost of a DDS storage system, taking into account the reduction in synthesis cost despite the increase in sequencing costs. Overall, composite letter‐based DDS provides a promising solution for ultra‐high logical density and reduced costs with DDS.

However, composite letter‐based DDS presents severe challenges. In the whole DDS process, there are many steps such as synthesis, replication, storage, and sequencing which may induce nucleotide errors.^[^ ^4^ ^]^ As composite letters are formed by combinations of four standard DNA nucleotides, these combination frequencies are vulnerable to nucleotide errors and severe sampling bias.^[^ ^3^ ^]^ This can result in high letter error rates, making error correction a major challenge for composite DDS.

To correct errors, the DDS system typically utilizes added information redundancy, including physical redundancy and logical redundancy.^[^ ^5^ ^]^ Traditional physical redundancy‐based methods duplicate the DNA molecules, expecting every piece of information to correctly appear in the majority of copies while decoding. This approach only corrects random errors. The state‐of‐the‐art DDS systems^[^ ^6^ ^]^ predominantly employ logical redundancy in the form of error‐correcting code (ECC)^[^ ^7^ ^]^ to address errors during DDS processes, correcting both random and systematic errors. ECC adds redundant information to the original data through mathematical algorithms, enabling error detection and correction. Grass et al.^[^ ^6^ ^]^ were the first to apply ECC in the realm of DDS, utilizing Reed‐Solomon (RS) code in a concatenated manner that enabled the correction of not only single‐nucleotide errors but also the loss of oligos. Erlich et al.^[^ ^6^ ^]^ combined RS code with fountain code – a type of ECC that theoretically approaches Shannon capacity for high information density. Press et al.^[^ ^6^ ^]^ developed a novel ECC system called HEDGES recently which demonstrated efficacy in correcting insertion and deletion errors. However, all these ECC systems suffer from the trade‐off between the added redundancy and logical density. A recent study^[^ ^8^ ^]^ capitalized on the specific error characteristics of nucleotides in the standard four nucleotide‐based DDS channel, combined with ECC to implement soft‐decision decoding in DDS.^[^ ^9^ ^]^ This approach Derrick^[^ ^8^ ^]^ greatly enhanced error correction capabilities without compromising logical density. In composite letter‐based DDS, previous attempts to correct composite letter errors used RS code^[^ ^10^ ^]^ and fountain code,^[^ ^11^ ^]^ without considering the letter characteristics. This resulted in the necessity of a sequencing depth of over 300× for accurate information recovery, compared to a mere 5×^[^ ^5^ ^]^ required for standard four‐nucleotide encoding, leading to significant increases in sequencing costs.

**Figure 1:**
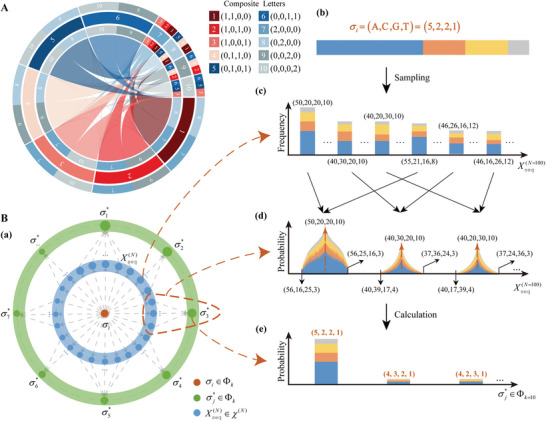
The core principles of Derrick‐cp decoding algorithm. A) A graphical representation of the letter transition library for k=2 and a sampling depth of 10. The concentric circle consists of ten sectors. Each sector describes the composite letter uncertainty, with three tracks. The middle track is the main track, representing the letter error rate. The inner track represents a transition outward, while the outer track represents a transition inward. Notably, for a given letter in the middle track, the corresponding letters in both the inner and outer tracks are almost identical. B) The schematic diagram (a) and corresponding example (b–e) of letter inference and transition library constructing strategies. In the schematic diagram, (b) the central orange point $\sigma_{i}$ represents an original letter, and the first set of arrows from the orange point $\sigma_{i}$ indicate the process of synthesis, storage, and sequencing, (c) resulting in observed frequencies at a specific sampling depth N, represented by the blue points $X_{\mathrm{seq}}^{\left(N\right)}$. Assuming a sampling depth of N, there are $C_{N+3}^{N}$ different combinations of observed frequencies, each with a distinct probability of occurrence, which is reflected in the varying sizes of the blue points in the blue layer. (d) The second set of arrows from the blue layer illustrates the process of letter inference, clustered by the closest letter represented by green points $\sigma_{j}^{*}$. (e) By calculating the proportion of each cluster, the transition probability from $\sigma_{i}$ to $\sigma_{j}^{*}$ in k is obtained. Overall, beginning with the central orange point $\sigma_{i}$, the process culminates in the outermost green point $\sigma_{j}^{*}$, representing the completion of the transition library construction. The size of the green points reflects the probability of transition from $\sigma_{i}$ to $\sigma_{j}^{*}$.

To address this, we propose an innovative soft‐decision decoding algorithm for composite letter‐based DDS, termed Derrick‐cp. This algorithm integrates letter error characteristics with ECC decoding, taking advantage of the variations in error sensitivity among letters which is the inherent letter heterogeneity arising from the simple encoding through combination proportion. For instance (**Figure** **1**A), in a ten‐letter composite alphabet, a letter represented by the combination proportion (1,1,0,0) is obviously more prone to divergence compared to another letter represented by (2,0,0,0). We calculated the letter error probability and transition probability by analyzing the impact of nucleotide errors and sampling bias on the composite letters, aiming to predict and correct letter errors. Derrick‐cp works by first inferring the closest composite letter from the observed frequencies, then predicting the inferred composite letter's error probability and transition probability, and generating predicted alternative letter sets for error correction. These sets are iterated for soft decision decoding, with the results verified in the final step.

Our proposed Derrick‐cp algorithm was tested through a comparative analysis with existing datasets and simulated experiments. The results indicated a significant reduction in sequencing depth required for full recovery, and overall cost savings of up to ≈22% for composite DNA‐based storage systems, compared with the previous study employing a hard decision decoding approach.^[^ ^3^ ^]^ In conclusion, the Derrick‐cp algorithm presents a reliable and cost‐effective solution for composite DNA‐based storage systems.

## 2 Results

### 2.1 Principle and Overview of Derrick‐cp Algorithm

The definition of composite DNA letter aligns with the previous study^[^ ^3^ ^]^ that a composite letter is a representation of a position in a sequence that constitutes a mixture of all four standard DNA nucleotides in a specified predetermined ratio $\sigma =(\sigma_{A},\sigma_{C},\sigma_{G},\sigma_{T})\;$ where $k=\sigma_{A}+\sigma_{C}+\sigma_{G}+\sigma_{T}$ is defined as the resolution parameter of the composite letter. When reading a message coded using composite letters at a certain sequencing depth, each composite position constitutes a mixture of all four standard DNA nucleotides, denoted as observed frequencies. Due to sampling bias and nucleotide errors, the observed frequencies will typically not precisely match any letter from the original alphabet.

Thereby, the first step in the decoding process is to identify the most probable original letter that generates the observed frequencies after sequencing and sampling. This step involves solving a Maximum A Posteriori (MAP) probability problem. Subsequently, RS code is applied to identify and correct any remaining errors. If the number of unknown errors in an RS(N,K) block does not exceed (N−K)/2, successful error correction can be achieved using hard decision decoding. Here, N represents the total length of the RS block, K represents the length of the uncoded original information, and (N−K) represents the length of redundancy. However, if the number of unknown errors exceeds (N−K)/2, soft decision decoding techniques need to be employed. Albeit the letter inference method is robust, there remains a small possibility that the number of errors in the inferred letter may exceed (N−K)/2.

Error positions can be easily predicted by comparing the Euclidean distances between the inferred letter and observed frequencies. However, predicting the corresponding correction values is challenging due to the complex composition of observed frequencies, which include four nucleotide bases with various types of errors such as insertions, deletions, and substitutions. To address this challenge, we make sense of error sensitivity among letters first by analyzing letter uncertainty with information entropy, and then construct the Bayesian model based on the introduced errors to quantify the letter uncertainty. In information theory, entropy can measure the uncertainty of information, and the smaller the entropy, the smaller the uncertainty and the less randomness.^[^ ^12^ ^]^ It clearly explains why a letter represented by the combination proportion (1,1,0,0) has a higher error rate than a letter represented by (2,0,0,0) in a ten‐letter composite alphabet (Figure 1A). That is, the uncertainty of the composite letter varies and arises from the specific compositions of the composite letters. Further, we construct a Bayesian model utilizing the occurrence frequency of the observed from a certain letter, to create a transition library including the letter error probability and transition probability, supporting the DDS process.

**Figure 2:**
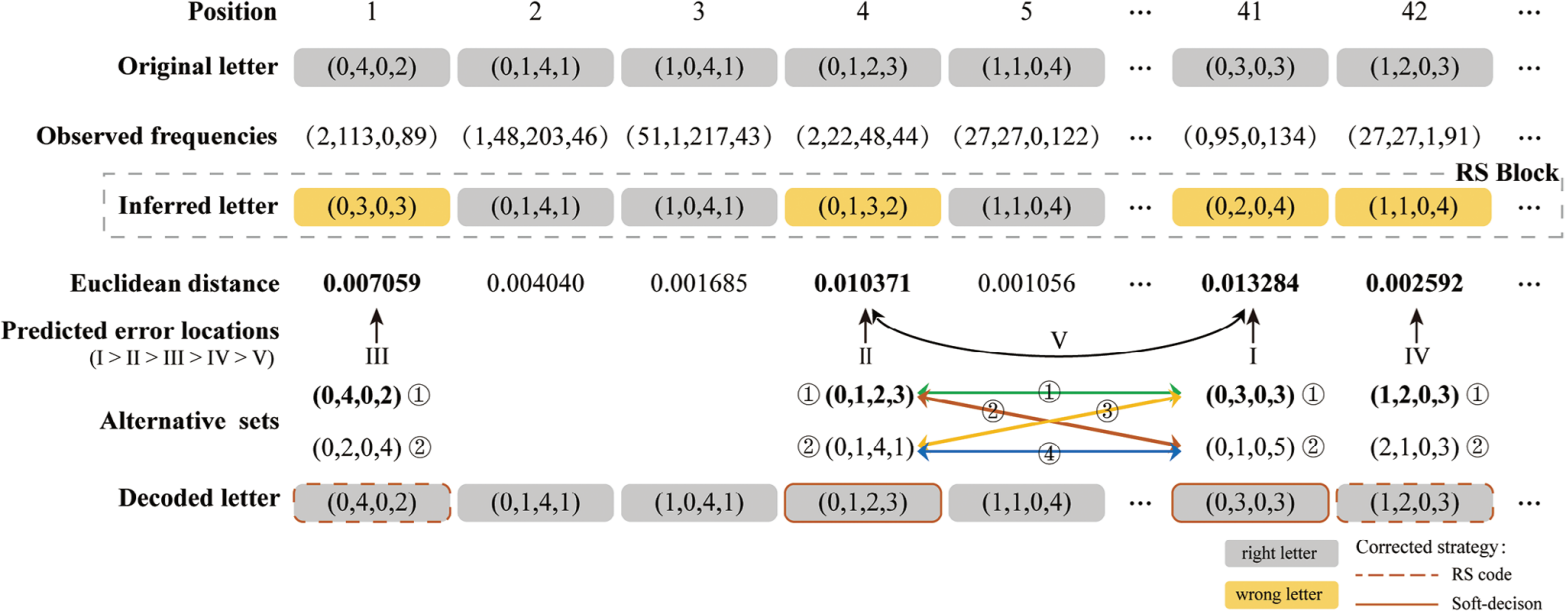
The decoding processes with an example of decoding an RS (45, 41) block. The soft decision decoder operates through four steps. A) Employs MAP probability to infer the composite letter based on the observed base frequencies. B) It assesses the reliability of the inferred letter by measuring the Euclidean distance between the observed frequencies and the inferred letter. C) It generates an alternative letterset for amending the previously inferred letter. D) It iterates the predicted error positions and corresponding values for soft decision decoding. The iterating process begins by attempting to correct a single position. In this example, there are four potential error positions, each with two alternative values. The iteration follows a specific order determined by reliability sorting, I>II>III>IV>V. Subsequently, the algorithm proceeds to correct two positions and the number of candidate error positions increased by one in the algorithm, when correcting one position decoding fails. When correcting two positions, there are six possible combinations of positions. The figure showcases the decoding paths and their sequential order when positions 4 and 41 are for correction, represented as: ①>②>③>④. The remaining errors are corrected by the RS code, which is within the correction capability of the RS code.

The soft decision decoder operates through four steps (**Figure** **2**): First, it infers the composite letter from the observed base frequencies through the MAP probability conditioned on the observation. Next, it measures the reliability of the inferred letter through the Euclidean distance between the observed frequencies and the inferred letter to predict the error positions. Third, it calls letter transition probabilities in the transition library: Due to the letter uncertainty, errors can cause the original letter to transform into other letters. The algorithm computes the probabilities of such transitions to provide alternative correction values for erroneous letters. Last, it iterates the predicted error positions and corresponding alternative correction letter sets to perform soft decision decoding. Each step of the algorithm follows specific principles, as described below.

### 2.2 Infer the Composite Composite Letter

**Figure 3:**
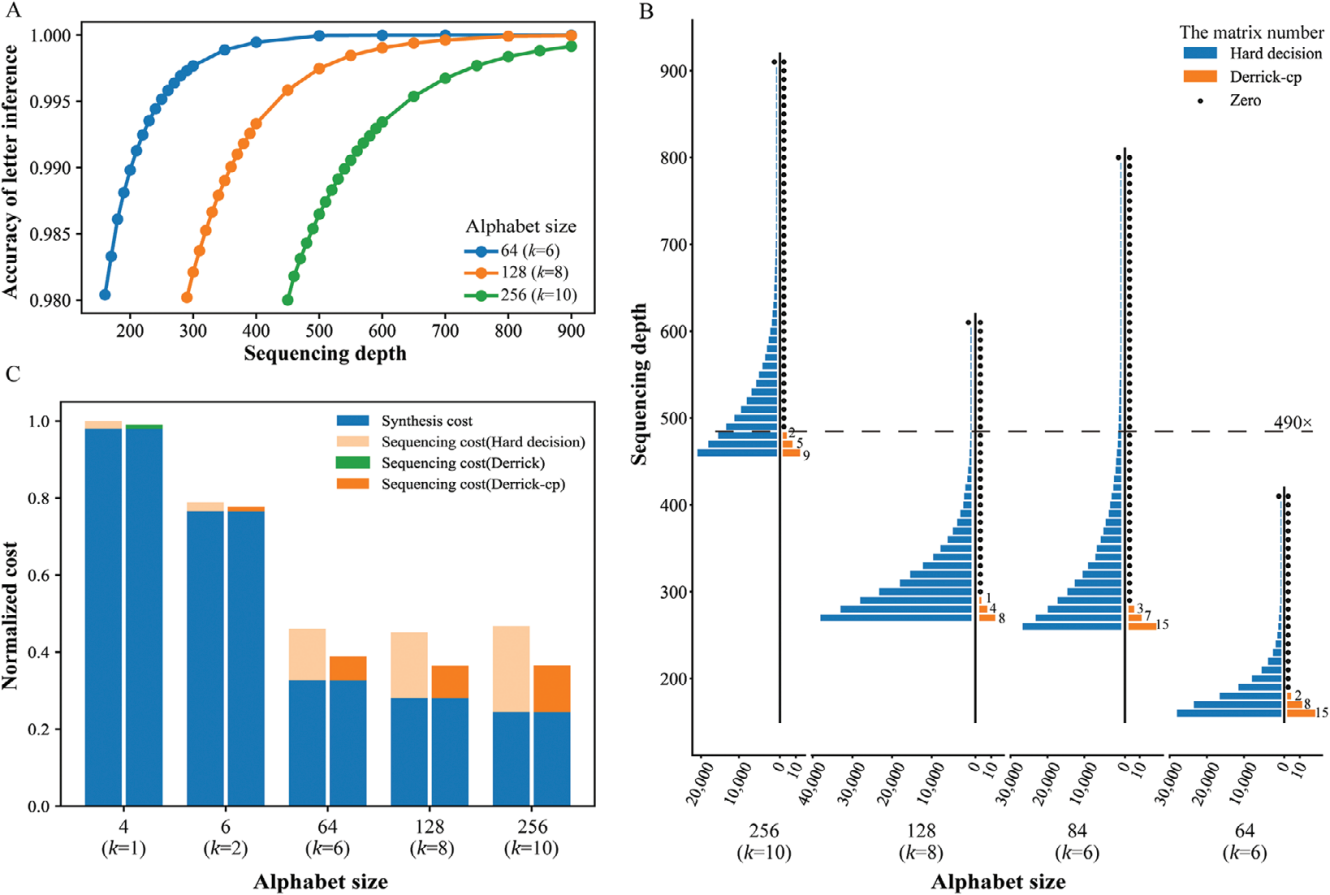
Decoding performance of Derrick‐cp algorithm in simulation tests. A) The accuracy of letter inference utilizing the MAP probability model. In a composite DNA‐based storage system using different alphabets, within the sequencing depth that the Derrick‐cp can decode with 100% accuracy, the letters Inference accuracy can reach 98%. B) Comparison of Derrick‐cp and hard‐decision decoding by the number of failed matrices with different alphabet tests. The horizontal axis represents the alphabet size, while the vertical axis represents the sequencing depth. The blue blocks indicate the failed matrices by utilizing the hard‐decision decoding strategy, while the orange blocks represent the adoption of the soft‐decision decoding strategy. Notably, the horizontal line labeled “490×” indicates that with the implementation of soft‐decision decoding, data encoded with an alphabet size of 256 can still be recovered with 100% accuracy. Conversely, with hard‐decision decoding, only data encoded with a reduced alphabet size of 64 can be recovered with the same level of accuracy. C) Comparison of overall costs of DDS systems employing different alphabets and decoding strategies. The horizontal axis represents the alphabet size, while the vertical axis represents the normalized cost. In this context, the costs are standardized by normalizing them to the total cost of a standard DDS system that utilizes four standard nucleotides and employs hard‐decision decoding.

To infer composite letters from observed frequencies, we first convert the sequencing readout to a vector of base frequencies and then calculate the Bayesian probability of each composite letter in the alphabet conditioned on the observed vector (Figure 1B). By utilizing this probability calculation, we select the letter with the MAP probability as the inferred letter. Moreover, the MAP inference model is designed to take into account the influence of bias and nucleotide errors introduced in the synthesis, storage, sampling, and sequencing steps of the process (Experimental Section). To improve the efficiency of the computational process, we formulate the probability function by considering the vector of letters as the independent variable and the Bayesian probability as the dependent variable. We derive the probability function and determine the vector point(s) that satisfy the maximum value of the function. About 3–5 composite letters in the vicinity of the extremum are then selected as alternatives, and their MAP probabilities are computed to choose the inferred letter. In our tests, the accuracy of letter inference is above 97.6% utilizing our approach (**Figure** **3**A, **Table** **1**, and Tables S1–S3).

**Table 1:** Decoding performance of Derrick‐cp algorithm in in vitro tests conducted with the alphabet size of 6.

Sequencing depth [×]	15	16	17	18	19	20	21	22	23	24	25	30
Accuracy												
Letter inference	0.9833	0.9839	0.9890	0.9900	0.9907	0.9932	0.9975	0.9984	0.9985	0.9990	0.9991	0.9996
Positions prediction	0.9708	0.9736	0.9693	0.9673	0.9698	0.9653	0.9470	0.9667	0.9444	0.8889	0.9983	1.0000
True letters prediction	0.9999	1.0000	0.9998	1.0000	1.0000	1.0000	1.0000	1.0000	1.0000	1.0000	1.0000	1.0000
Probability												
Decoder error	0.0182	0.0168	0.0055	0.0046	0.0035	0.0015	0.0001	0.0000	0.0000	0.0000	0.0000	0.0000
Correct decoder error	0.9693	0.9714	0.9815	0.9821	0.9909	0.9894	0.9333	0.0000	0.0000	0.0000	0.0000	0.0000

The “Accuracy” category represents the performance of the Derrick‐cp algorithm decoding process, including letter inference accuracy and error prediction accuracy. The “Probability” category showcases the occurrence probability of RS decoder error in our tests and the probability of successfully detecting and accurately decoding the RS blocks with the decoder error using the Derrick‐cp algorithm.

### 2.3 Locate the Error Positions and Predict the Corresponding True Letters

During the decoding of an RS block, after the inference step, Derrick‐cp identifies error positions based on letter reliability which is evaluated by calculating the Euclidean distance between the observed vector and the inferred letter vector. To facilitate this calculation, the vectors are first normalized, and positions with the maximum distance are selected as candidate error positions (Experimental Section). Subsequently, we predict the true values for these candidate positions (see Figure S1, Supporting Information). Albeit our letter inference step is robust, it may still leave a certain proportion of errors. This is due to original letters potentially being misidentified as other letters. To address this issue, we construct a Bayesian model that describes the transitions of composite DNA letters from one to another, taking into account errors that may occur during the synthesis, sequencing, and sampling processes (Experimental Section). Using the Bayesian model, we create a transition library that provides alternative letters for each letter transition (Figure 1A). This library is constructed by selecting the greater probability for each letter transition, thus forming alternative letter sets for letter correction at each predicted position. Throughout the error correction process, we iteratively select alternative letter sets from the transition library to ultimately correct the errors.

### 2.4 Iterate Prediction Sets for RS Soft Decision Decoding

Due to the imperfection of error prediction, the set of predicted error positions and true letters inevitably contains false positives. However, the RS decoder can judge the correctness of the predictions participating in the soft‐decision process, as any incorrect prediction can introduce more errors and cause soft‐decision strategy futile and decoding failure. Thus, our method sorts the prediction sets, containing the candidate error position set and the corresponding alternative letter sets, by the letter reliability and transition probability, and then uses an iterative algorithm to attempt subsets one by one until RS correction succeeds or all subsets have been tried. Larger prediction sets offer greater chances of gathering all the errors, but require higher computational costs of searching feasible subsets. Thus, with this trade‐off, Derrick‐cp improves the error‐correcting capability of RS code by sacrificing the computation efficiency to some extent and is capable of solving any data block with any number of errors with unlimited computational resources. In practice, Derrick‐cp limits iterative counts for each data block using a threshold and gives up blocks beyond this threshold.

### 2.5 Detect RS Decoder Error

When errors surpass the hard‐decision error correction threshold, RS decoder errors^[^ ^13^ ^]^ frequently arise, despite the highly reliable error‐correction results of RS codes. In such cases, the erroneous message is perceived by the decoder as a correctable error, but the error is mistakenly amended to an incorrect message. To tackle this challenge, the Derrick‐cp algorithm employs a two‐pronged approach to detect the RS decoder errors and perform recorrecting. First, the algorithm examines the decoded information of each RS block by checking if the rectified letter is present in the transition library corresponding to the current position. The transition library contains the most probable alternative letters for the inferred letter at that position. If the rectified letter is not found in the transition library, it indicates a potential error in the RS decoding process. In such cases, the current block is flagged as a decoder error and undergoes re‐decoding until the decoding result contains the rectified letters that are present in the corresponding error positions' transition libraries. Secondly, the integrity of the entire matrix is verified using a Cyclic Redundancy Check (CRC) code with a 32‐bit checksum to ensure the accuracy of the complete matrix, which consists of ten consecutive blocks (as depicted in Figure S2, Supporting Information) after all the blocks have been decoded by the RS decoder.^[^ ^14^ ^]^ If any error is detected, the algorithm returns to the previously flagged blocks and re‐decodes them again until they pass the CRC32 detection. The transition library verification method enables the rapid and precise identification of erroneous blocks in the presence of RS decoder errors, without the need for additional redundancy, despite the relatively high occurrence rate of RS decoder error (up to 5.44%).

### 2.6 Derrick‐cp Decreases Sequencing Depth for Decoding and Overall Costs

To validate the effectiveness of soft‐decision decoding on real‐world composite DNA‐based storage systems, we encoded a message from Leon et al.^[^ ^3^ ^]^ using two samples with primer designs “AC” and “GT”, with file sizes of 2.12 MB and 6.42 MB, respectively. The six‐letter composite alphabet used was $\sum_{6}=\left\{A,C,G,T,M,K\right\}$, where M=(1,1,0,0) and K=(0,0,1,1). Our error correction scheme utilized both an RS code and a CRC32 for data integrity. Specifically, we designed our system to use a CRC32 checksum for every 10 RS code blocks contained in a matrix. The sequencing readout was sampled and decoded through Derrick‐cp. Compared with hard‐decision decoding, our soft‐decision decoding reduced the required sequencing depths for successful recovery by approximately one‐fold in both sample tests (**Table** **2**).

**Table 2:** The result comparison by decoding approaches in vitro tests was conducted with the alphabet size of 6.

Sequencing depth [×]	15	16	17	18	19	20	21	22	23	24	25	30
Hard decision	8784	8214	3569	3042	2423	1105	76	27	11	4	5	1
Derrick‐cp	8	1	0	0	0	0	0	0	0	0	0	0

#Matrix = 25 475

The table displays the result comparison of Derrick‐cp and hard‐decision decoding approaches by the number of failed matrices. The “#Matrix” indicates the total number of matrices used in each decoding test.

To comprehensively evaluate and demonstrate the robustness of our approach, we expanded our study by considering larger k values (k=6,8,10) and conducted simulations using larger datasets. The datasets were generated from 20.8 MB files including book, html, et al. To represent the larger *k* values, we selected composite alphabet sizes of 64 ($\sum_{64}$), 128 ($\sum_{128}$), and 256 ($\sum_{256}$) for k=6, k=8, and k=10, respectively. These sizes were chosen based on their efficient and convenient conversion between binary information and composite DNA letters. To ensure better distinguishability among the selected subset of letters in the composite alphabet, we carefully considered the letter error rates and transition probabilities between different letters, and selected those with a relatively low error rate. Increasing the value of k resulted in an increased depth requirement for successful decoding.

However, our soft‐decision decoding approach significantly reduced the required sequencing depth for each k (Figure 3B). Specifically, Derrick‐cp reduced the required sequencing depth from 900× to 490× for tests with the alphabet size of 256 compared with hard‐decision decoding, from 600× to 300× with the alphabet size of 128, from 800× to 290× with the alphabet size of 84, and from 400× to 190× with alphabet size of 64. Moreover, for RS (45,41), the hard‐decision decoding had a correction capability of 2 errors. In contrast, our experiment utilizing the soft‐decision decoding increased the correction capability to 6, tripling the error correction capability.

We also calculated the total cost reduction achieved by reducing the sequencing redundancy required, compared to previous studies^[^ ^3^ ^]^ (Table S5, Supporting Information). Our results demonstrate that, compared to the standard nucleotide‐based DDS system, as exemplified by Leon et al.’s study^[^ ^3^ ^]^ which achieved an overall cost reduction of ≈52% by using composite letters, our study further reduces the overall cost to ≈63.58% by incorporating soft‐decision decoding (Figure 3C) based that a moderate ratio of the synthesis cost to sequencing cost ratio ($C_{\mathrm{syn}}:C_{\mathrm{seq}}$) is 1000:1.

### 2.7 Derrick‐cp Increases Logical Density for Large Composite Alphabets

Based on our simulation experiments, we investigated the decoding performance of different k values in composite DNA‐based storage. We found that soft‐decision decoding allowed for successful decoding of the information even with larger k values, compared to hard‐decision decoding, at the same error rate. For instance, when the sequencing depth was 490×, hard‐decision decoding could only correct the information encoded with the composite alphabet $\sum_{64}$ for k=6 while soft‐decision decoding successfully recovered the information with the composite alphabet $\sum_{256}$ for k=10 (Figure 3B). This resulted in 1.33 times increase in information density compared to hard‐decision decoding. Additionally, our approach enables reliable exploitation of the full composite alphabet without the need to select a subset previously required for accurate decoding in some studies. For example, when using k=6 and a full alphabet size of 84 with Derrick‐cp instead of a subset size of 64, all files could be recovered at lower sequencing depths (Figure 3B).

### 2.8 Factors Related to the Performance of Derrick‐cp Algorithm

The core of soft‐decision decoding lies in locating the error positions and predicting the corresponding true values. By utilizing the predicted true values obtained from alternative letter sets, Derrick‐cp directly corrects the errors by replacing the previous letters, necessitating both the accuracy of the positions and the correctness of the letters. Our results demonstrate that the accuracy of these candidate error positions is 97.15%, and the prediction accuracy of true letters is 99.88%, as illustrated in Table 1 and Tables S1–S3 (Supporting Information).

Moreover, we studied the effect of time limits on the performance of our decoding algorithm by analyzing running time distributions of matrices. Our results showed that over 99.46% of matrices were completed within 1 s, while only 0.06% took more than a specified longer duration (e.g., 100s) (Table S4, Supporting Information). This finding indicates that a relatively short time limit is sufficient for solving the majority of matrices in composite DNA‐based storage systems using our proposed Derrick‐cp algorithm.

## 3 Discussion

Composite letters enabled DDS with significantly high logical density. However, the inherent letter heterogeneity in the alphabet makes them susceptible to errors during DDS processes, thereby posing challenges for accurate information retrieval. Additionally, the high costs associated with DDS have hindered its widespread adoption, necessitating cost‐reduction measures. To address these challenges, we have introduced the Derrick‐cp algorithm. The key innovations of the Derrick‐cp algorithm lie in its utilization of the inherent letter heterogeneity arising from the simplified design in letter through combination proportion, constructing a probability model that predicts letters from observed frequencies and enables the prediction and correction of letter errors, and consequently, facilitating soft‐decision decoding in composite DDS. Indeed, Derrick‐cp exploits the heterogeneity among letters to effectively predict and correct errors in DDS systems using composite letters, which is not feasible for DDS systems using four standard nucleotides. On the other hand, Derrick is specifically conceived for the DDS systems using the four standard nucleotides. It only focuses on addressing nucleotide errors, such as substitutions, insertions, and deletions. Derrick, when creating consensus DNA sequences from sequencing reads, utilizes a general prediction model that errors occurring at high‐copy sequencing sites will exhibit lower consensus across multiple sequences, aiding in error prediction and correction.

The effectiveness of the Derrick‐cp algorithm was demonstrated through extensive simulations and in vitro experiments. Derrick‐cp enhanced the error correcting capability by the number of correctable errors, for RS (45,41), from 2 with hard‐decision decoding to 6. Our results indicated a significant reduction in sequencing depth by one‐fold and overall costs by up to 22% for composite DNA‐based storage systems, while maintaining 100% accuracy of decoding.

While the synthesis of composite DNA letters presents technological challenges, such as the necessity for modifications in the design of synthesis hardware, the work by Takahashi et al.^[^ ^15^ ^]^ has indeed showcased the viability of constructing a flexible synthesis system. Moreover, through our consultation with Beijing Genomics Institute, we have gained encouraging insights into advancements in composite DNA synthesis. They have achieved success in developing specialized chips for composite DNA synthesis. These chips are specifically designed to accommodate small‐size alphabets, such as k=2 or k=3. The availability of specialized synthesis chips tailored for composite DNA synthesis demonstrates ongoing efforts to optimize the synthesis process and potentially reduce associated costs.

Several strategies can be employed to further enhance Derrick‐cp's decoding performance in future studies: First, our current algorithm treats each position on a sequence as independent without considering potential influences from neighboring positions. However, incorporating correlations and dependencies between neighboring positions could provide valuable contextual information and improve decoding accuracy. Second, our current inference model and the Bayesian model focus on overall error, but do not differentiate between specific error types. Developing models that can distinguish and account for different error types, such as insertions, deletions, or substitutions,^[^ ^4^, ^16^ ^]^ could lead to more precise and tailored error correction. By targeting specific error types, future studies can design algorithms and strategies that effectively address the unique challenges associated with each type of error.

Composite letter‐based DDS with Derrick‐cp provides a complementary option for DDS in more refined applications. Composite letters, formed by mixing the standard four nucleotides in a combination ratio, expand the possible space of combinations compared with the standard four nucleotides. From the perspective of entropy in information theory,^[^ ^12^, ^17^ ^]^ this combination increases the overall entropy of the system. However, this expansion of possibilities compromises the system's stability, making it more susceptible to errors. For practical applications, the appropriate method can be chosen based on the importance of data and the desired storage scale. For large‐scale data storage with a certain degree of error tolerance, such as for storing large media files like movies and videos, composite letter‐based DDS shows potential. When considering data storage demands that prioritize accuracy and long‐term stability, the traditional four‐nucleotide DNA storage method is preferable. For data storage that prioritizes accuracy and long‐term stability, such as government archival of important documents or preservation of historical artifacts and cultural heritage data, the traditional four‐nucleotide DNA storage method is more suitable.

In conclusion, our study highlights the potential of integrating MAP probability estimation with soft‐decision decoding for enhancing accuracy and affordability in composite letter‐based storage systems. We believe Derrick‐cp will hopefully contribute to the development and potential applications of composite letter‐based DDS.

## 4 Experimental Section

### Inference of Composite DNA Letters

Due to errors in DNA synthesis, sequencing, and sampling processes, the observed frequencies might not precisely match any letter from the original alphabet. In this study, we employ Bayesian posterior probability to perform accurate identification of the original composite letter from the observed read counts.

First, we define a composite DNA alphabet following by previous study,^[^ ^3^ ^]^ represented by

(1) $$\Phi_{k}=\left\{\left(\sigma_{A},\sigma_{C},\sigma_{G},\sigma_{T}\right):\sigma_{i\in \left\{A,C,G,T\right\}}\in Z_{\geq 0},\sum_{i\in \left\{A,C,G,T\right\}}\sigma_{i}=k\right\}$$

where $\sigma =(\sigma_{A},\sigma_{C},\sigma_{G},\sigma_{T})\;$ denotes a composite letter and k is a tunable parameter that represents the resolution of the composite alphabet. The size of the composite DNA alphabet is given by $|\Phi_{k}|\;=C_{k+3}^{k}$.

The observed read counts are defined as:

(2) $$X_{\mathrm{seq}}^{\left(N\right)}\left(\sigma \right)\;=\;\left(X_{A}\left(\sigma \right),X_{C}\left(\sigma \right),X_{G}\left(\sigma \right),X_{T}\left(\sigma \right)\right)$$

and

(3) $$\begin{array}{ccc} & & X_{\mathrm{seq}}^{\left(N\right)}\in X^{\left(N\right)}=\left\{\left(X_{A},X_{C},X_{G},X_{T}\right):X_{i\in \left\{A,C,G,T\right\}}\in Z_{\geq 0},\sum_{i\in \left\{A,C,G,T\right\}}X_{i}=N\right\} \end{array}$$

To infer the original composite letter σ from the observed $X_{\mathrm{seq}}^{\left(N\right)}$, we define $P(\sigma |X_{\mathrm{seq}}^{\left(N\right)})$ as the conditional probability of composite letter σ given the observed vector $X_{\mathrm{seq}}^{\left(N\right)}$. The Inference of the original composite letter involves estimating the maximum likelihood value $\sigma^{*}\in \Phi_{k}$ that maximizes $P(\sigma |X_{\mathrm{seq}}^{\left(N\right)})$, denoted as the function $f(X_{\mathrm{seq}}^{\left(N\right)})$.

(4) $$f\left(X_{\mathrm{seq}}^{\left(N\right)}\right)=\sigma^{*}=\mathrm{argma}x_{\sigma \in \Phi_{k}}\;\left(P\left(\sigma |X_{\mathrm{seq}}^{\left(N\right)}\right)\right)$$

The posterior probability of σ: $P(\sigma |X_{\mathrm{seq}}^{\left(N\right)})\;$ is calculated using Bayes' theorem as follows, where the probability of σ occurring: P(σ) is modeled as a prior distribution, the probability of sampled vector $X_{\mathrm{seq}}^{\left(N\right)}$: $P(X_{\mathrm{seq}}^{\left(N\right)})$ are modeled as posterior distribution and $P(X_{\mathrm{seq}}^{\left(N\right)}|\sigma )$ is the probability of observation $X_{\mathrm{seq}}^{\left(N\right)}$ given the encoded composite letter σ.

(5) $$P\left(\sigma |X_{\mathrm{seq}}^{\left(N\right)}\right)\;=\frac{P\left(X_{\mathrm{seq}}^{\left(N\right)}|\sigma \right)\;P\left(\sigma \right)}{P\left(X_{\mathrm{seq}}^{\left(N\right)}\right)}\;,\;\sigma \in \Phi_{k}$$

Noted that the values of $P\left(\sigma \right)\left(\sigma \in \Phi_{k}\right)$ and $P(X_{\mathrm{seq}}^{\left(N\right)})$ are the same for every composite letter $\sigma \in \Phi_{k}$, thus the calculation of $f(X_{\mathrm{seq}}^{\left(N\right)})$ can be simplified.

The conditional probability $P(X_{\mathrm{seq}}^{\left(N\right)}|\sigma )$ is calculated by

(6) $$P\;\left(X_{\mathrm{seq}}^{\left(N\right)}|\sigma \right)=\frac{P\left(X_{\mathrm{seq}}^{\left(N\right)}\cap \sigma \right)}{P\left(\sigma \right)}\;,\;\sigma \in \Phi_{k}$$

Noted that the value of P(σ) is the same for every composite letter $\sigma \in \Phi_{k}$, thus the calculation of $P(X_{\mathrm{seq}}^{\left(N\right)}|\sigma )$ can be simplified.

(7) $$P\;\left(X_{\mathrm{seq}}^{\left(N\right)}\cap \sigma \right)=\frac{C\left(A\right)}{C\left(B\right)}\;=\frac{C\left(X_{\mathrm{seq}}^{\left(N\right)}\cap \sigma \right)}{\sum_{X_{\mathrm{seq}}^{\left(N\right)}\in X^{\left(N\right)}}C\left(X_{\mathrm{seq}}^{\left(N\right)}\right)}$$

where C represents the occurring counts. Noted that the C(B) is the same for every composite letter $\sigma \in \Phi_{k}$, $f(X_{\mathrm{seq}}^{\left(N\right)})$ can be further simplified.

Assuming independence of the nucleotide errors and sampling bias, C(A) follow the binomial distribution, represented as:

(8) $$C\left(A\right)=\;C\left(X_{\mathrm{seq}}^{\left(N\right)}\cap \sigma \right)=C_{F\left(\sigma \right)}^{X_{\mathrm{seq}}^{\left(N\right)}}=\prod_{i\in \left\{A,C,G,T\right\}}C_{F_{i}\left(\sigma \right)}^{X_{i}\left(\sigma \right)}$$

where F(σ) is the total sample counts in the general process of sampling, containing 1% of nucleotide errors,^[^ ^16^ ^]^ represented as:

(9) $$\begin{array}{ccc} F_{i}\left(\sigma \right) & = & f*\frac{\sigma_{i}}{k}*\left(1-P_{\mathrm{error}}\right)+\frac{P_{\mathrm{error}}}{3}*\left(1-\frac{\sigma_{i}}{k}\right)*f,\\ f & = & 0.5\mathrm{fmol},\;i\in \left\{A,C,G,T\right\} \end{array}$$

Based on the above formula derivations and analysis, the infer of the most likely original composite letter by $f(X_{\mathrm{seq}}^{\left(N\right)})$ can be can be simplified as:

(10) $$f\left(X_{\mathrm{seq}}^{\left(N\right)}\right)=\sigma^{*}=\mathrm{argma}x_{\sigma \in \Phi_{k}}\;\left(\prod_{i\in \left\{A,C,G,T\right\}}C_{F_{i}\left(\sigma \right)}^{X_{i}\left(\sigma \right)}\right)$$

By taking the derivative of the above equation and selecting the proportion for which the derivative equals zero, we can find the value that maximizes the function. However, the resulting proportion may not necessarily be a standard proportion σ. Therefore, we choose the standard proportion that is closest to the extremum as the desired $\sigma^{*}$. Typically, we select 3–5 alternatives for consideration.

### Generation of Alternative Letter Sets for Soft Decision Decoding

To generate alternative letter sets from observed read count vectors for soft decision decoding, we calculate transition probabilities from other composite DNA letters to the inferred letter and sort them in descending order. This alternative letter set is then selected iteratively and sent to the RS decoder for error correction. Transition probabilities between letters are calculated based on a Bayesian model describing how composite DNA letters can transition from one to another due to errors during synthesis, sequencing, and sampling processes.

To estimate transition probabilities, we first calculate the likelihoods of all possible observed vectors starting from a certain composite letter under a given sequencing depth. Then, Bayesian inference methods are used to infer the closest composite letter from the observed vector, resulting in a transition probability library for each letter containing the most likely correct letters based on their proximity to the current letter (Figure 1A). More details are provided in the Supporting Information.

### Derrick‐cp Decoding Algorithm

In this study, we propose a novel Derrick‐cp decoding algorithm that combines Bayesian inference with RS soft decision decoding to accurately identify composite DNA letters from observed read count frequencies and enhance error‐correcting performance. The decoding algorithm consists of the following well‐defined steps.

1. Composite DNA letter inference: For each position within an RS block, infer the composite DNA letter based on observed read count frequencies using Bayesian inference.
2. Hard decision decoding: Decode the inferred letters with the RS decoder. If decoding is unsuccessful, proceed to soft decision decoding.
3. Soft decision decoding:
  - Candidate position set and alternative letter set generation: The positions in the candidate position set are selected by the Euclidean distance between the observed frequencies and the inferred letter following normalization. For each candidate error position, an alternative letter set is created, prioritizing the letters according to the descending order of transition probabilities from other composite letters to the inferred letter at this selected position.
  - Decoding path formulation: Incrementally increase the number of candidate error positions and generate combinations of these positions for formulating decoding paths. For each error position combination, apply modifications using the corresponding alternative letter set in order of transition probability from highest to lowest.
  - Iterative decoding: Iterate through decoding paths and enter the RS decoder for error correction until achieving successful decoding or exceeding the upper error limit.
4. Decoder error detection and validation: After successful RS code decoding, inspect potential decoder errors by comparing corrected letters with transition libraries. If a corrected letter is absent from libraries, mark it as potentially experiencing a decoder error.
5. Matrix recovery verification: Perform CRC32 check on the entire matrix to verify information recovery. If unsuccessful, revisit RS blocks marked as decoder error for another round of soft decision decoding. Repeat steps 3–5 until the matrix is successfully decoded and passes the CRC32 check.

## Conflict of Interest

The authors declare no conflict of interest.

## Author Contributions

Y.X. and L.D. contributed equally to this work. J.R. conceived the study; Y.X., J.R., L.D., and S.W. designed the algorithms. Y.X. implemented the algorithms. L.D., J.R., and Y.X. wrote and revised the manuscript. Y.X. and L.D. contributed to the data simulation. All authors reviewed the final manuscript.

## Supporting information

Supporting Information

## Data Availability

The previously published data for in vitro experiments was downloaded from the European Nucleotide Archive (ENA) under accession PRJEB32427. The Derrick‐cp source code and dataset for simulation tests are hosted by GitHub at: https://github.com/xu‐yaping/Derrick‐cp.
