# Supplementary material for: Overcoming the High Error Rate of Composite DNA Letters‐Based Digital Storage through Soft‐Decision Decoding
Source: Adv Sci (Weinh). 2024 Jun 14;11(30):2402951. doi: 10.1002/advs.202402951 (PMC11321706; doi:10.1002/advs.202402951)
Supplement: Supplementary file 1 — Supporting Information [file ADVS-11-2402951-s001.docx]

**Title**

**Overcoming the High Error Rate of Composite DNA Letters-Based Digital Storage through Soft-Decision Decoding**

*Yaping Xu^†^, Lulu Ding^†^, Shigang Wu, and Jue Ruan**

Y. Xu, L. Ding, S. Wu, J. Ruan

Shenzhen Branch, Guangdong Laboratory of Lingnan Modern Agriculture, Genome Analysis Laboratory of the Ministry of Agriculture and Rural Affairs, Agricultural Genomics Institute at Shenzhen, Chinese Academy of Agricultural Sciences

7 Pengfei Street Dapeng New District, Shenzhen 518120, P. R. China

E-mail: [ruanjue@caas.cn](mailto:ruanjue@caas.cn)

L. Ding

National Engineering Laboratory for Big Data System Computing Technology, Shenzhen University

Shenzhen 518060, PR China

† These authors contributed equally to this work.

* Corresponding authors.

The file includes Supplementary Notes, Figures S1–S7 and Tables S1–S5.

**Supporting Information**

**Subsampling.** In order to obtain an appropriate sample size for decoding, we perform subsampling prior to the decoding process. Subsampling is the process of selecting a subset of sequencing reads from the total sequenced sample at a specified sequencing depth. This step is crucial for reducing computational complexity and ensuring the accuracy of our proposed decoding algorithm.

To carry out subsampling, we first estimate the required sequencing depth based on factors such as the original data size, composite letter resolution, error rates in DNA synthesis and sequencing processes, and desired accuracy level for decoding. We then randomly select a subset of reads from the total sequenced sample corresponding to the determined sequencing depth. This subsampled dataset is subsequently used as input for our decoding algorithm, enabling us to efficiently recover the original information while minimizing errors introduced during DNA synthesis, sequencing, and sampling processes.

**A multinominal model for the composite DNA letters.** We construct a multinomial model to analyze the distribution of observed read count vectors $X_{\mathrm{seq}}^{\left( N \right)}$ for composite letters. The count vectors are generated by sequencing and counting $N$ independent molecules. To account for the effects of various factor on the observed read counts, we consider the following parameters: synthesis bias, sampling bias, synthesis error, base degradation, and sequencing error. Synthesis and sampling bias affect the original proportional distribution, and are represented by $\left\{ P_{\mathrm{bias}} \right\}_{\sigma\to\sigma^{'}}$, where $\sigma$ and $\sigma'$ are composite letters in $\Phi_{k}$. Synthesis error, base degradation and sequencing error affect the molecules through specific types of errors, such as deletion, insertion, and mismatch, and the rates of these errors are represented by $\left\{ P_{\mathrm{error}} \right\}_{i\to j}$ , where $i$ and$j$are letters in $\left\{ A,C,G,T \right\}$.

$$\begin{aligned} P_{\mathrm{bias}}=\left\{ P_{\mathrm{syn}} \right\}_{\sigma\to\sigma^{'}}+\left\{ P_{\mathrm{sam}} \right\}_{\sigma\to\sigma^{'}}, \sigma,\sigma^{'}\in\Phi_{k}\#\left( 1 \right) \end{aligned}$$

$$\begin{aligned} P_{\mathrm{error}}=\left\{ P_{\mathrm{syn}} \right\}_{i\to j}+\left\{ P_{\deg} \right\}_{i\to j}+\left\{ P_{\mathrm{seq}} \right\}_{i\to j} , i,j\in\left\{ A,C,G,T \right\}\#\left( 2 \right) \end{aligned}$$

We assume independence of the different error sources and incorporate all errors into a generalized multinomial model with slightly altered probabilities. The corrected probability vector $p(\sigma)=\left( \rho_{A}\left( \sigma\right), \rho_{T}\left( \sigma\right),\rho_{C}\left( \sigma\right),\rho_{G}\left( \sigma\right) \right)$ is used in the multinomial model to account for the effects of the bias and error parameters. The model can be expressed as:

$$\begin{aligned} X_{\mathrm{seq}}^{\left( N \right)} \left( \sigma, P_{\mathrm{bias}}, P_{\mathrm{error}} \right)\sim\mathrm{Multinomial}\left( N, p\left( \sigma\right) \right), X_{\mathrm{seq}}^{\left( N \right)}\epsilon\mathcal{X}^{\left( N \right)}\#\left( 3 \right) \end{aligned}$$

**Calculate transitional probability.** For each $X_{\mathrm{seq}}^{\left( N \right)}$ in $\mathcal{X}^{\left( N \right)}$, based on the formula $C\left( X_{\mathrm{seq}}^{\left( N \right)} | \sigma\right)$ and $f\left( X_{\mathrm{seq}}^{\left( N \right)} \right)$, we can calculate the occurrence frequency of $X_{\mathrm{seq}}^{\left( N \right)}$ from all $\sigma$ in $\Phi_{k}$, and obtain the optimal letter $\sigma^{*}$ of the observed $X_{\mathrm{seq}}^{\left( N \right)}$ which is categorized into $S\left（ \sigma^{*} \right）$. The $S\left（ \sigma^{*} \right）$ contain the $X_{\mathrm{seq}}^{\left( N \right)}$ the optimal letter of which is $\sigma^{*}$, defined as:

$$\begin{aligned} S\left（ \sigma^{*} \right）=\left\{ X_{\mathrm{seq}}^{\left( N \right)}\in\mathcal{X}^{\left( N \right)}, f\left( X_{\mathrm{seq}}^{\left( N \right)} \right)=\sigma^{*} \right\}\#(4) \end{aligned}$$

According to the $S\left（ \sigma^{*} \right）$ and $C\left( X_{\mathrm{seq}}^{\left( N \right)} | \sigma\right)$, we can calculate the ratio $P^{\left( N \right)}\left( \sigma^{*} | \sigma\right)$ transitioning from $\sigma$ to $\sigma^{*}$.

$$\begin{aligned} P^{\left( N \right)}\left( \sigma^{*} | \sigma\right)=\frac{O\left( \sigma^{*} | \sigma\right)}{\sum_{\sigma^{*}\in\Phi_{k}} O\left( \sigma^{*} | \sigma\right)},\sigma^{*},\sigma\epsilon\Phi_{k}\#(5) \end{aligned}$$

where $O\left( \sigma^{*} | \sigma\right)$ represents the sum of occurrence frequency of the each $X_{\mathrm{seq}}^{\left( N \right)}$ in $S\left（ \sigma^{*} \right）$, defined as:

$$\begin{aligned} O\left( \sigma^{*} | \sigma\right)=\sum_{X_{\mathrm{seq}}^{\left( N \right)}\in S\left（ \sigma^{*} \right）} C\left( X_{\mathrm{seq}}^{\left( N \right)} | \sigma\right)\#\left( 6 \right) \end{aligned}$$

In short, given the known parameters $k$ and $N$, we can calculate the transition probability $P^{\left( N \right)}\left( \sigma^{*} | \sigma\right)$ from $\sigma$ to $\sigma^{*}$ by $C\left( X_{\mathrm{seq}}^{\left( N \right)} | \sigma\right)$. And the transition probability will play a pivotal role in soft-decision process. In detail, the transition probabilities between the same composite letter mean the right probability of the composite letter, by which we can select the composite letter with high accuracy for DNA Storage. And we can filter out the composite letter pairs with the transition probability to construct the transition library which can correct errors efficiently in soft-decision process.

**Encoding scheme**. A composite DNA letter represents a position in a sequence that consists of a mixture of all four standard DNA nucleotides in a specified predetermined ratio $\sigma=\left( \sigma_{A}, \sigma_{C}{, \sigma}_{G}, \sigma_{T} \right)$, where $k= \sum_{i\in\left\{ A, C,G,T \right\}} \sigma_{i}$ is defined as the resolution parameter of the composite letter. A composite DNA alphabet is a set of composite DNA letters, typically sharing a common resolution $k$. The full composite alphabet of resolution $k$, denoted as $\Phi_{k}$ is the set of all $\sigma=\left( \sigma_{A}, \sigma_{C}{, \sigma}_{G}, \sigma_{T} \right)$ and consists of $C_{k+3}^{k}$ composite letters. The distance between different letters varies.

The encoding process involves converting files into oligos by splitting original files into matrices and encoding with Cyclic Redundancy Check (CRC) codes with a 32-bit checksum and Reed-Solomon (RS) codes before mapping to composite letters. The CRC32 code is added at the end of each matrix, and RS code is encoded with each column of the matrix. Then, the composite sequence is generated in each row by mapping to encoded composite letters. In our experiments with the composite alphabet $\sum_{6}$ for $k=2,$ encoding, we mapped every 5 bits into 2 neighboring composite DNA positions resulting in a composite DNA sequence with a length of 151 nucleotides (Figure S3). The encoding process using the composite alphabet $\sum_{64},\sum_{84},\sum_{128},\sum_{256}$ for $k=6$, $k=8$ and$k=10$, were depicted in Figures S4-S7.


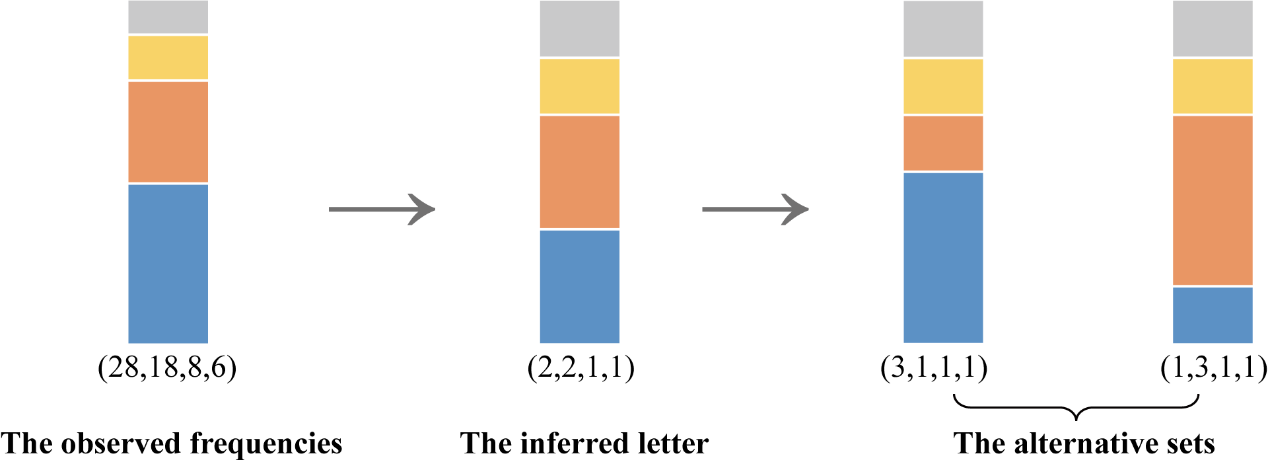


**Figure S1. An example of decoding a single synthesized composite position.** The decoding process includes obtaining the inferred letter and its alternative sets. The observed frequencies are used to infer the source, $\sigma= (2,2,1,1)$, as the closest composite letter, using Maximum A Posteriori probability (Experimental Section); And in the decoding process of Derrick-cp, each inferred letter has one alternative set from the transition library, which can predict the corresponding true values and amend the inferred letter.


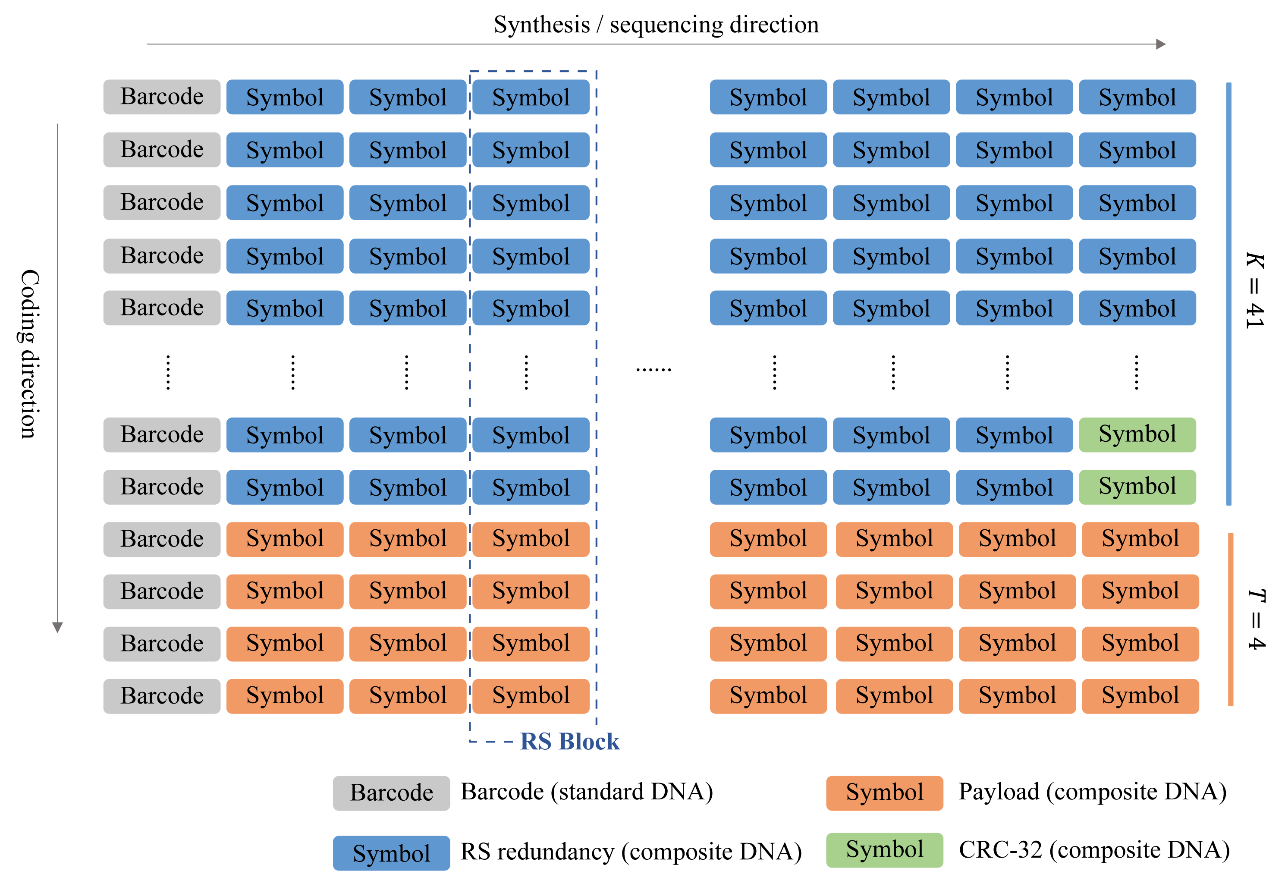
**Figure S2.** **The CRC matrix is the decoding unit of Derrick-cp**. The CRC matrix comprise 10 continuous RS blocks and is encoded with a CRC32 code. And the CRC32 code can validate the accuracy of the complete CRC matrix after all RS blocks have been decoded by the RS decoder.


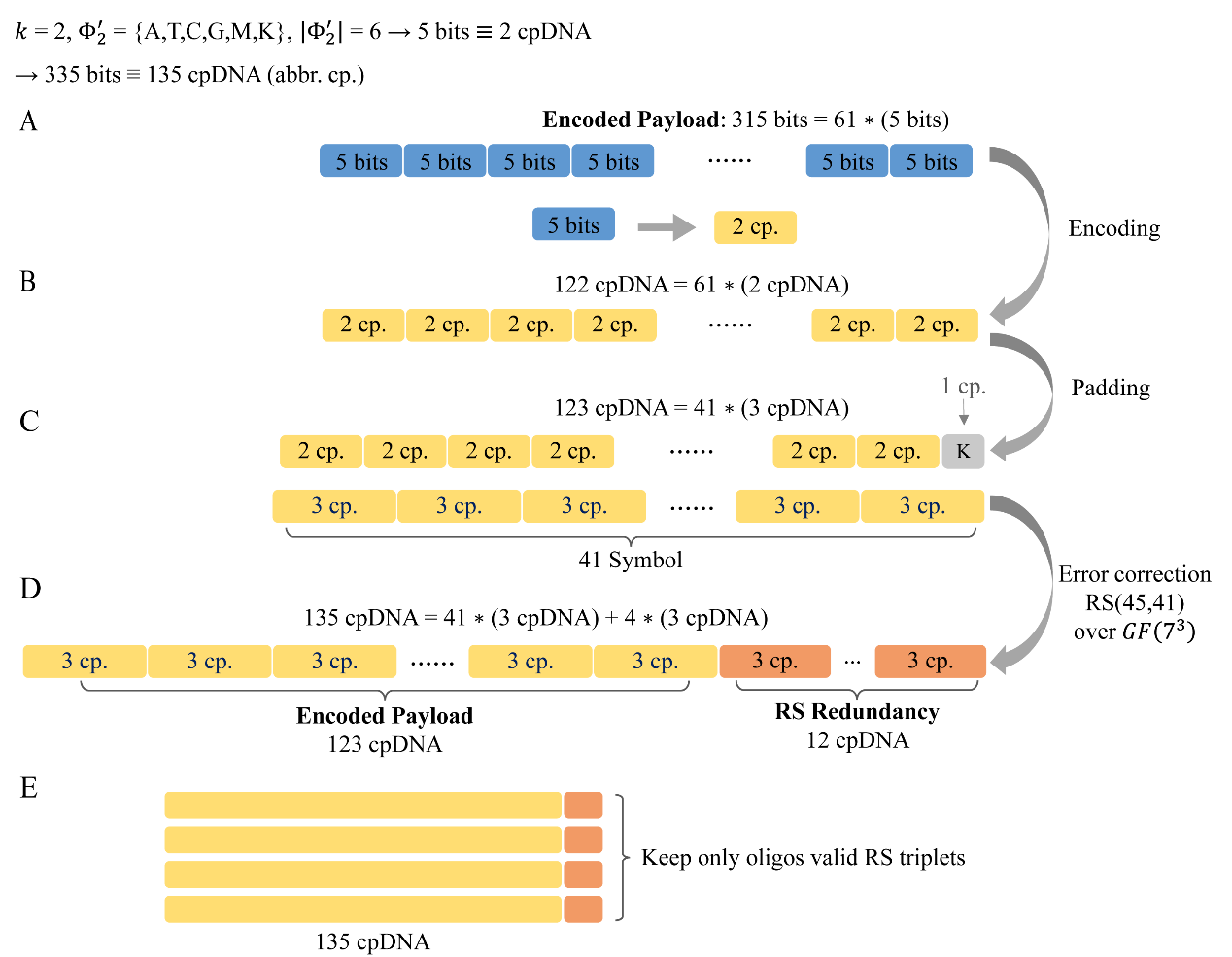
**Figure S3. Detailed encoding scheme of a 6-letter composite DNA based storage system.**

**A.** The message was cut to chunks of 315 bits.

**B.** The 315 bits payload was converted into a 122nt of 6-letter composite alphabet by converting every 5 bits to 2 composite letters.

**C.** The composite message was padded to 123nt by adding a "K" at the end.

**D.** The 129nt payload is encoded to 135nt by using a RS (45,41) RS over $GF(7^{3})$.

**E.** The encoded oligos are filtered so that only oligos in which all 6 redundancy letters are within $\sum_{6}$ are kept. The desired number of oligos are kept and sent to synthesis.


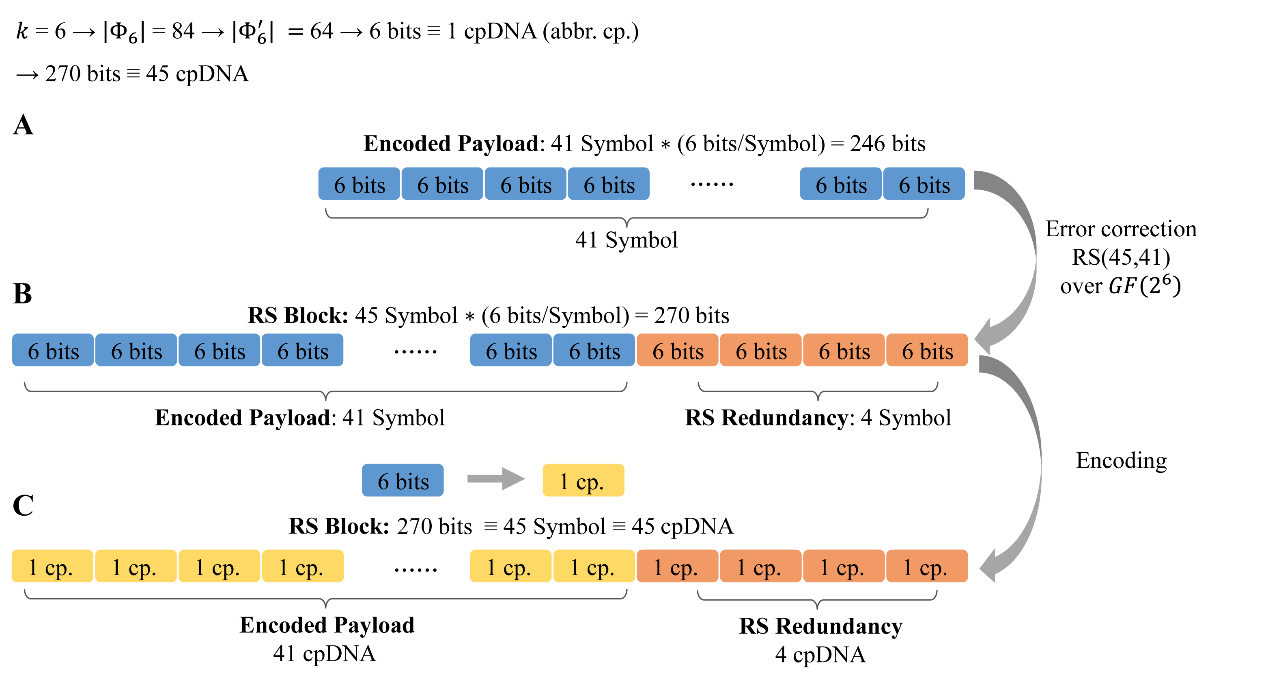
**Figure S4. Detailed encoding scheme of a 64-letter composite DNA based storage system.**

**A.** The message was cut to chunks of 246 bits, and the payload was converted into 41 symbols. Each symbol comprises 6 bits, which can form 64 types of symbols.

**B.** The payload with 41 symbols was encoded to 45 symbols by using a RS (45,41) RS over $GF(2^{6})$, shaping one RS block.

**C.** The RS block with 45 symbols was converted into a 45nt of 64-letter composite alphabet by converting every symbol to 1 composite letter.

**
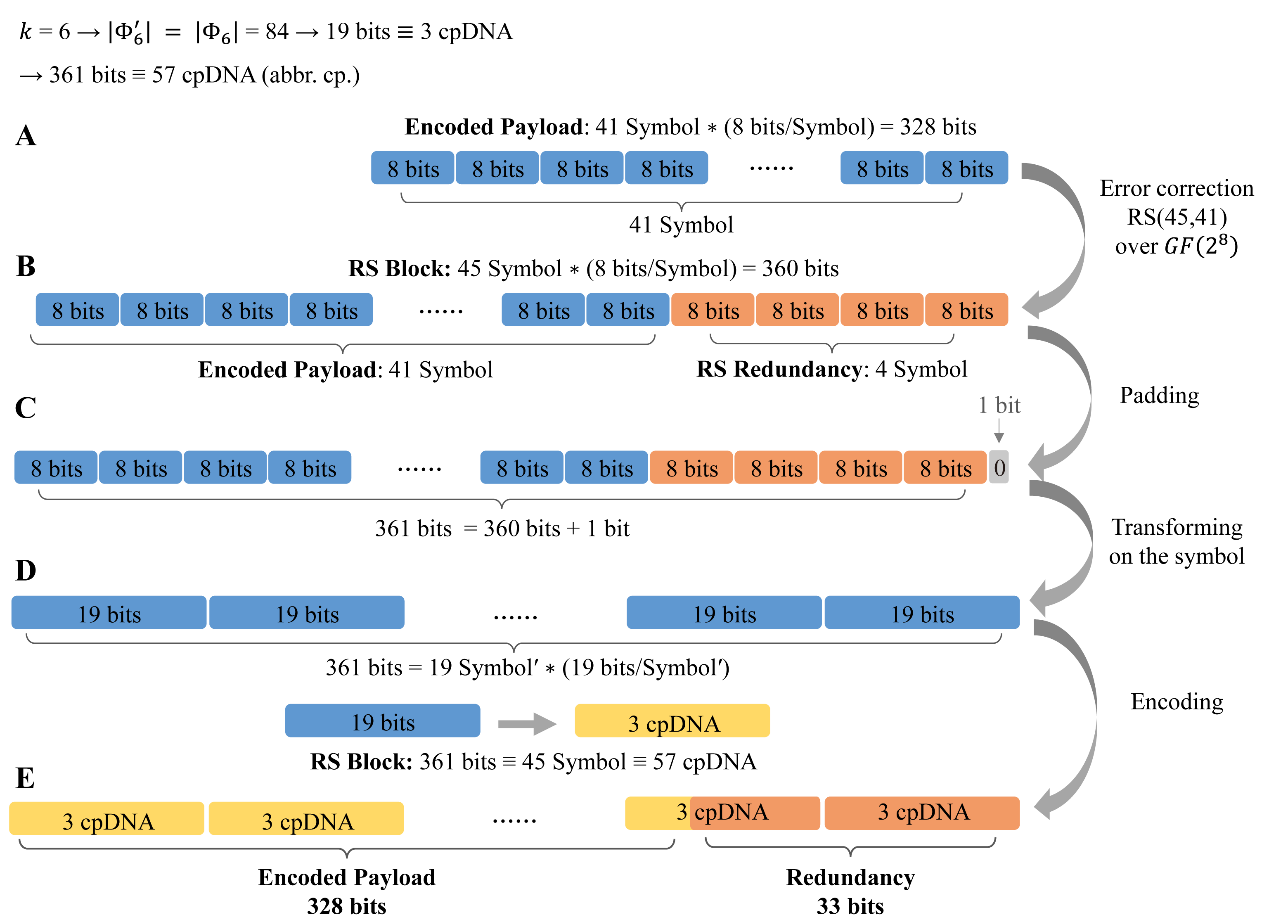
Figure S5. Detailed encoding scheme of an 84-letter composite DNA based storage system.**

**A.** The message was cut to chunks of 328 bits, and the payload was converted into 41 symbols. Each symbol comprises 8 bits.

**B.** The payload with 41 symbols was encoded to 45 symbols by using a RS (45,41) RS over $GF(2^{8})$, shaping one RS block.

**C.** The RS block was padded to 361 bits by adding a "0" at the end.

**D and E.** Every 19 bits are encoded into triads of letter positions, that is, the combination of 84, 84, and 75 letters. The RS block was encoded into 57nt of 84-letter composite alphabet.


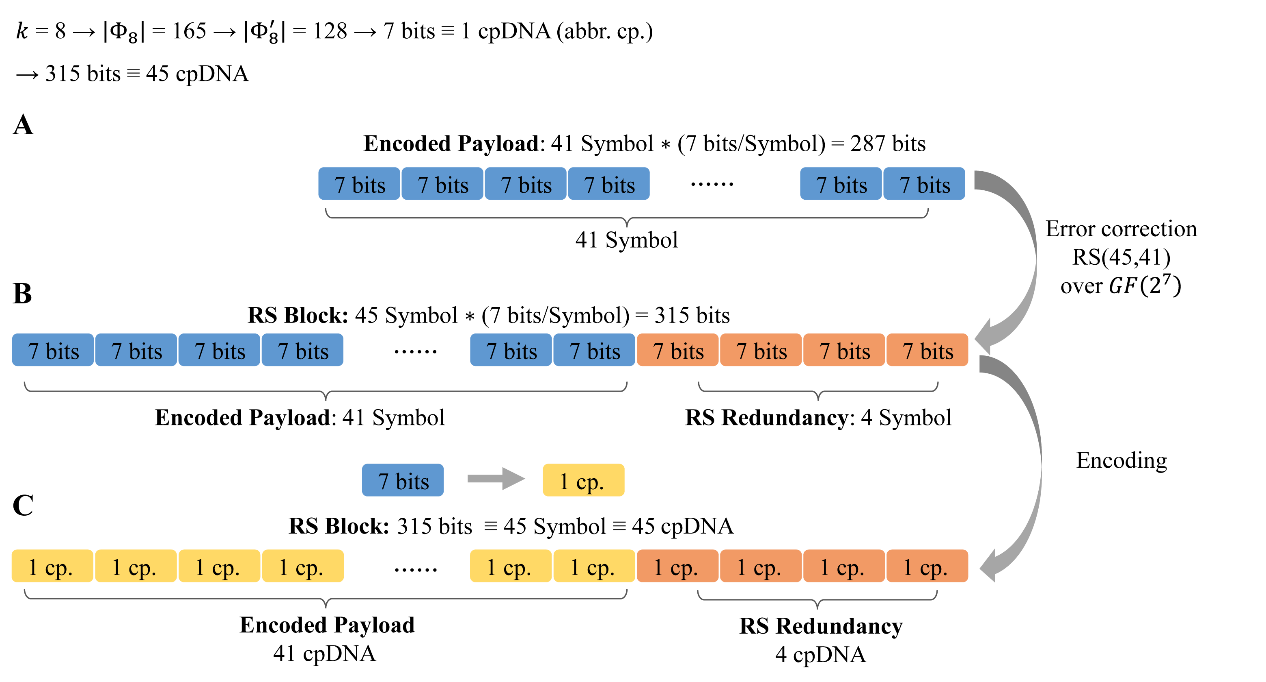
**Figure S6. Detailed encoding scheme of a 128-letter composite DNA based storage system.** Similar to Figure S4 with the following differences:

**A.** Each chunk is 287 bits and each symbol comprises 7 bits.

**B.** The payload is encoded using a RS (45,41) RS over $GF(2^{7})$.

**C.** The RS block with 45 symbols was converted into a 45nt of 128-letter composite alphabet by converting every symbol to 1 composite letter.


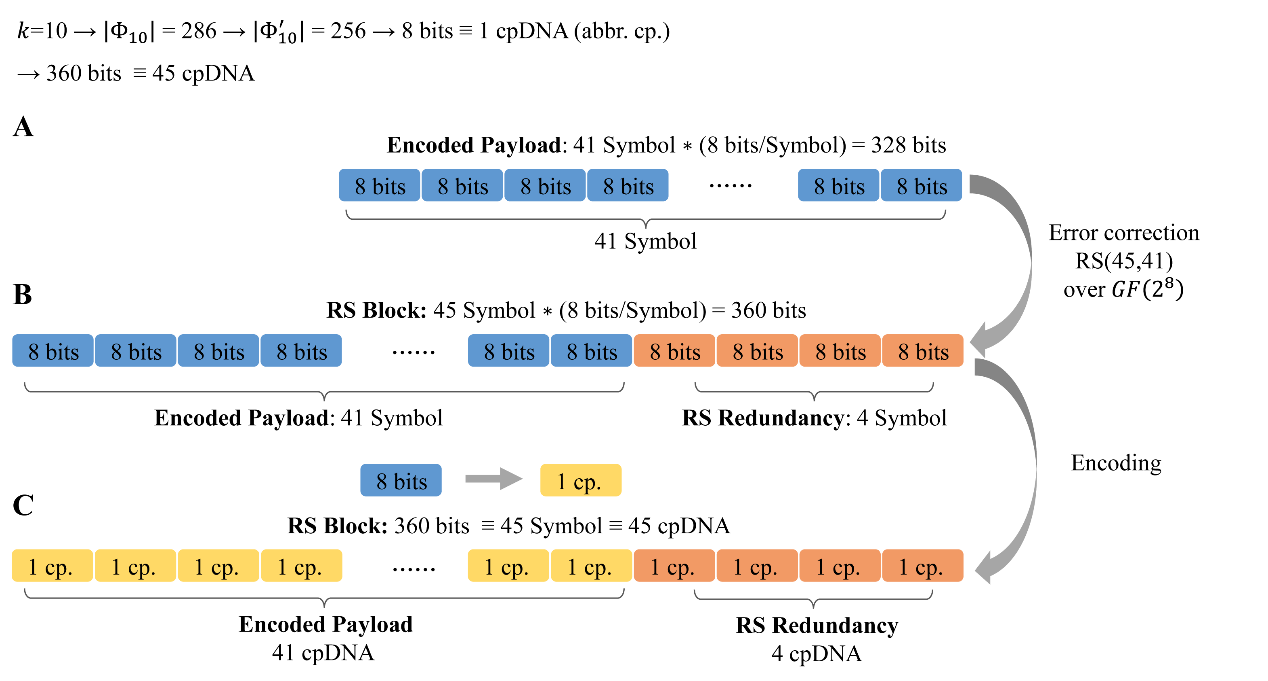
**Figure S7. Detailed encoding scheme of a 256-letter composite DNA based storage system.** Similar to Figure S4 with the following differences:

**A.** Each chunk is 328 bits and each symbol comprises 8 bits.

**B.** The payload is encoded using a RS (45,41) RS over $GF(2^{8})$.

**C.** The RS block with 45 symbols was converted into a 45nt of 256-letter composite alphabet by converting every symbol to 1 composite letter.

**Table S1. Decoding performance in different strategy and the related influencing factors of Derrick-cp.**

| **Sequencing depth [×]** | **160** | **170** | **180** | **190** | **200** | **220** | **240** | **250** | **300** | **350** | **400** |
| --- | --- | --- | --- | --- | --- | --- | --- | --- | --- | --- | --- |
| **Strategy** |  |  |  |  |  |  |  |  |  |  |  |
| Hard decision | 30402 | 23476 | 16525 | 11485 | 7869 | 3549 | 1373 | 1035 | 137 | 18 | 3 |
| Derrick-cp | 15 | 8 | 2 | 0 | 0 | 0 | 0 | 0 | 0 | 0 | 0 |
| **Accuracy** |  |  |  |  |  |  |  |  |  |  |  |
| Letter inference | 0.9804 | 0.9833 | 0.9861 | 0.9881 | 0.9898 | 0.9925 | 0.9944 | 0.9952 | 0.9977 | 0.9989 | 0.9995 |
| Positions prediction | 0.9475 | 0.9574 | 0.9652 | 0.9711 | 0.9770 | 0.9830 | 0.9893 | 0.9909 | 0.9952 | 1.0000 | 1.0000 |
| True letters prediction | 0.9998 | 0.9998 | 0.9999 | 0.9999 | 0.9999 | 1.0000 | 1.0000 | 1.0000 | 1.0000 | 1.0000 | 1.0000 |
| **Probability** |  |  |  |  |  |  |  |  |  |  |  |
| Decoder error | 0.0544 | 0.0348 | 0.0223 | 0.0140 | 0.0090 | 0.0035 | 0.0014 | 0.0009 | 0.0001 | 0.0000 | 0.0000 |
| Correct decoder error | 0.9851 | 0.9887 | 0.9919 | 0.9940 | 0.9947 | 0.9984 | 0.9940 | 1.0000 | 1.0000 | 0.0000 | 0.0000 |

#Matrix = 69990

The "Strategy" category shows the performance comparison of the Derrick-cp decoding with hard-decision decoding by the number of failed matrices. The "Accuracy" and "Probability" category show the factors related to the performance of Derrick-cp algorithm. Similar to Table 1, except that the table is based on the encoding scheme of a 64-letter composite DNA in $k=6$.

**Table S2. Decoding performance in different strategy and the related influencing factors of Derrick-cp.**

| **Sequencing depth [×]** | **270** | **280** | **290** | **300** | **320** | **340** | **350** | **400** | **450** | **500** | **600** |
| --- | --- | --- | --- | --- | --- | --- | --- | --- | --- | --- | --- |
| **Strategy** |  |  |  |  |  |  |  |  |  |  |  |
| Hard decision | 38054 | 33005 | 28023 | 23221 | 15414 | 9619 | 7790 | 2031 | 520 | 119 | 6 |
| Derrick-cp | 8 | 4 | 1 | 0 | 0 | 0 | 0 | 0 | 0 | 0 | 0 |
| **Accuracy** |  |  |  |  |  |  |  |  |  |  |  |
| Letter inference | 0.9759 | 0.9781 | 0.9802 | 0.9821 | 0.9852 | 0.9879 | 0.9890 | 0.9933 | 0.9959 | 0.9975 | 0.9990 |
| Positions prediction | 0.9492 | 0.9547 | 0.9596 | 0.9647 | 0.9724 | 0.9780 | 0.9810 | 0.9901 | 0.9937 | 0.9972 | 1.0000 |
| True letters prediction | 1.0000 | 1.0000 | 1.0000 | 1.0000 | 1.0000 | 1.0000 | 1.0000 | 1.0000 | 1.0000 | 1.0000 | 1.0000 |
| **Probability** |  |  |  |  |  |  |  |  |  |  |  |
| Decoder error | 0.0769 | 0.0579 | 0.0423 | 0.0306 | 0.0159 | 0.0078 | 0.0058 | 0.0009 | 0.0002 | 0.0000 | 0.0000 |
| Correct decoder error | 0.9982 | 0.9928 | 0.9943 | 0.9947 | 0.9965 | 0.9981 | 0.9989 | 0.9949 | 1.0000 | 0.0000 | 0.0000 |

#Matrix = 61725

The "Strategy" category shows the performance comparison of the Derrick-cp decoding with hard-decision decoding by the number of failed matrices. The "Accuracy" and "Probability" category show the factors related to the performance of Derrick-cp algorithm. Similar to Table 1, except that the table is based on the encoding scheme of a 128-letter composite DNA in $k=8$.

| **Sequencing depth [×]** | **460** | **470** | **480** | **490** | **500** | **520** | **540** | **550** | **600** | **700** | **900** |
| --- | --- | --- | --- | --- | --- | --- | --- | --- | --- | --- | --- |
| **Strategy** |  |  |  |  |  |  |  |  |  |  |  |
| Hard decision | 20898 | 18034 | 15452 | 13338 | 11158 | 7915 | 5422 | 4699 | 1697 | 247 | 3 |
| Derrick-cp | 9 | 5 | 2 | 0 | 0 | 0 | 0 | 0 | 0 | 0 | 0 |
| **Accuracy** |  |  |  |  |  |  |  |  |  |  |  |
| Letter inference | 0.9818 | 0.9831 | 0.9843 | 0.9854 | 0.9865 | 0.9883 | 0.9899 | 0.9906 | 0.9934 | 0.9967 | 0.9992 |
| Positions prediction | 0.9714 | 0.9743 | 0.9774 | 0.9787 | 0.9796 | 0.9839 | 0.9868 | 0.9893 | 0.9932 | 0.9987 | 1.0000 |
| True letters prediction | 0.9977 | 0.9973 | 0.9972 | 0.9969 | 0.9966 | 0.9957 | 0.9950 | 0.9945 | 0.9929 | 0.9925 | 0.9971 |
| **Probability** |  |  |  |  |  |  |  |  |  |  |  |
| Decoder error | 0.0260 | 0.0200 | 0.0152 | 0.0117 | 0.0085 | 0.0049 | 0.0025 | 0.0021 | 0.0005 | 0.0000 | 0.0000 |
| Correct decoder error | 0.9992 | 0.9994 | 0.9994 | 0.9995 | 0.9993 | 0.9992 | 1.0000 | 1.0000 | 1.0000 | 0.0000 | 0.0000 |

**Table S3. Decoding performance in different strategy and the related influencing factors of Derrick-cp.**

#Matrix = 53940

The "Strategy" category shows the performance comparison of the Derrick-cp decoding with hard-decision decoding by the number of failed matrices. The "Accuracy" and "Probability" category show the factors related to the performance of Derrick-cp algorithm. Similar to Table 1, except that the table is based on the encoding scheme of a 256-letter composite DNA in $k=10$.

| $\boldsymbol{k=2(}\boldsymbol{\sum}_{\boldsymbol{6}}\boldsymbol{)}$ | | | | $\boldsymbol{k=6(}\boldsymbol{\sum}_{\boldsymbol{64}}\boldsymbol{)}$ | | | | $\boldsymbol{k=8(}\boldsymbol{\sum}_{\boldsymbol{128}}\boldsymbol{)}$ | | | | $\boldsymbol{k=10(}\boldsymbol{\sum}_{\boldsymbol{256}}\boldsymbol{)}$ | | | |
| --- | --- | --- | --- | --- | --- | --- | --- | --- | --- | --- | --- | --- | --- | --- | --- |
| **Depth [×]** | **Time [s]** | | | **Depth [×]** | **Time [s]** | | | **Depth [×]** | **Time [s]** | | | **Depth [×]** | **Time [s]** | | |
|  | **<1** | **<5** | **<100** |  | **<1** | **<5** | **<100** |  | **<1** | **<5** | **<100** |  | **<1** | **<5** | **<100** |
| 15 | 0.9896 | 0.9943 | 1 | 170 | 0.9919 | 0.9967 | 0.9995 | 270 | 0.9704 | 0.9884 | 0.9989 | 470 | 0.9902 | 0.9979 | 0.9995 |
| 16 | 0.9966 | 0.9977 | 1 | 180 | 0.9962 | 0.9983 | 0.9999 | 280 | 0.9808 | 0.9915 | 0.9991 | 480 | 0.9862 | 0.9908 | 0.9916 |
| 17 | 0.9974 | 1 | 1 | 190 | 0.9965 | 0.9983 | 1 | 290 | 0.9870 | 0.9944 | 0.9996 | 490 | 0.9958 | 0.9991 | 1 |
| 18 | 0.9968 | 0.9968 | 1 | 200 | 0.9981 | 0.9991 | 0.9995 | 300 | 0.9890 | 0.9953 | 0.9998 | 500 | 0.9889 | 0.9927 | 0.9940 |
| 19 | 0.9929 | 0.9929 | 1 | 210 | 0.9992 | 0.9998 | 1 | 310 | 0.9949 | 0.9970 | 0.9991 | 510 | 0.9904 | 0.9939 | 0.9951 |
| 20 | 1.0000 | 1.0000 | 1 | 220 | 0.9993 | 0.9995 | 0.9997 | 320 | 0.9969 | 0.9986 | 0.9998 | 520 | 0.9916 | 0.9932 | 0.9942 |
| 21 | 1 | 1 | 1 | 230 | 1 | 1 | 1 | 330 | 0.9976 | 0.9982 | 0.9999 | 530 | 0.9932 | 0.9942 | 0.9950 |
| 22 | 1 | 1 | 1 | 240 | 0.9978 | 0.9978 | 0.9978 | 340 | 0.9984 | 0.9987 | 0.9999 | 540 | 0.9972 | 0.9981 | 0.9985 |
| 23 | 1 | 1 | 1 | 250 | 1 | 1 | 1 | 350 | 0.9991 | 0.9995 | 1.0000 | 550 | 0.9959 | 0.9976 | 0.9982 |
| 24 | 1 | 1 | 1 | 300 | 1 | 1 | 1 | 400 | 1 | 1 | 1 | 600 | 0.9982 | 0.9982 | 0.9991 |
| 25 | 1 | 1 | 1 | 350 | 1 | 1 | 1 | 500 | 1 | 1 | 1 | 700 | 1 | 1 | 1 |
| 30 | 1 | 1 | 1 | 400 | 1 | 1 | 1 | 600 | 1 | 1 | 1 | 800 | 1 | 1 | 1 |

**Table S4. Running time (by matrix) distribution of Derrick-cp decoding algorithm on *in vitro* and simulated datasets.**

| **Study** | **Data**  **encoded** | **Synthesis** | **Error correcting** | **Sequencing platform** | **Sequencing coverage** | **Information density [bits/bp]** | **Normalized cost** |
| --- | --- | --- | --- | --- | --- | --- | --- |
| Church et al.^[^[^1^](#_ENREF_1)^]^ | 650 KB | Phosphoramodite | high coverage | Illumina | 3000× | 0.60 | 10.32 |
| Goldman et al.^[^[^2^](#_ENREF_2)^]^ | 630 KB | Phosphoramodite | multiply copies | Illumina | 51× | 0.19 | 8.56 |
| Grass et al.^[^[^3^](#_ENREF_3)^]^ | 80 KB | Phosphoramodite | RS+RS | Illumina | 372× | 0.86 | 2.47 |
| Bornholt et al.^[^[^4^](#_ENREF_4)^]^ | 151 KB | Phosphoramodite | Huffman code | Illumina | 128× | 0.57 | 3.06 |
| Blawat et al.^[^[^5^](#_ENREF_5)^]^ | 22 MB | Phosphoramodite | RS+BCH+CRC16 | Illumina | 160× | 0.89 | 2.02 |
| Erlich et al^.[^[^6^](#_ENREF_6)^]^ | 2 MB | Phosphoramodite | Fountain + RS | Illumina | 10.5× | 1.19 | 1.31 |
| Yazdi et al.^[^[^7^](#_ENREF_7)^]^ | 3 KB | Phosphoramodite | MSA | ONT | 200× | 1.71 | 1.09 |
| Organick et al.^[^[^8^](#_ENREF_8)^]^ | 33 KB | Phosphoramodite | RS+RS | ONT | 36× | 0.81 | 1.98 |
| Organick et al^.[^[^8^](#_ENREF_8)^]^ | 200 MB | Phosphoramodite | RS+RS | Illumina | 5× | 0.81 | 1.92 |
| Lee et al^.[^[^9^](#_ENREF_9)^]^ | 96 B | Enzymatic | RS+BCH | ONT | 175× | 1.57 | 1.16 |
| Chandak et al.^[^[^10^](#_ENREF_10)^]^ | 11 KB | Phosphoramodite | Convolution + RS | ONT | 14× | 0.56 | 2.80 |
| Meiser et al.^[^[^11^](#_ENREF_11)^]^ | 176 KB | Phosphoramodite | RS+RS | Illumina | 200× | 0.85 | 2.18 |
| Press et al.^[^[^12^](#_ENREF_12)^]^ | 2 MB | Phosphoramodite | Hash + RS | Illumina | 50× | 1.20 | 1.35 |
| Weigang et al.^[^[^13^](#_ENREF_13)^]^ | 37.8 KB | Phosphoramodite | LDPC | ONT | 16.8× | 1.19 | 1.32 |
| Derrick^[^[^14^](#_ENREF_14)^]^ | 5.2 MB | Phosphoramodite | RS+CRC64 | Illumina | 4× | 1.37 | 1.13 |
| Derrick[[^14^](#_ENREF_14)^]^ | 5.2 MB | Phosphoramodite | RS+CRC64 | ONT | 8× | 1.56 | 1.00 |
| Leon et al.^[^[^15^](#_ENREF_15)^]^ | 6.4 MB | Phosphoramodite | RS+Fountain | Illumina | ~40× | 1.96 | 0.82 |
| Derrick-cp | 6.4 MB | Phosphoramodite | RS+CRC32 | Illumina | 17× | 2.06 | 0.76 |

**Table S5. Comparison of information density and required sequencing coverage to prior work.**

**References**

[1] G. M. Church, Y. Gao, S. Kosuri, *Science* **2012**, *337* (6102), 1628, <https://doi.org/10.1126/science.1226355>.

[2] N. Goldman, P. Bertone, S. Chen, C. Dessimoz, E. M. LeProust, B. Sipos, E. Birney, *Nature* **2013**, *494* (7435), 77, <https://doi.org/10.1038/nature11875>.

[3] R. N. Grass, R. Heckel, M. Puddu, D. Paunescu, W. J. Stark, *Angewandte Chemie International Edition* **2015**, *54* (8), 2552, <https://doi.org/10.1002/anie.201411378>.

[4] J. Bornholt, R. Lopez, D. M. Carmean, L. Ceze, G. Seelig, K. Strauss, presented at *Proceedings of the Twenty-First International Conference on Architectural Support for Programming Languages and Operating Systems*, **2016**.

[5] M. Blawat, K. Gaedke, I. Hütter, X.-M. Chen, B. Turczyk, S. Inverso, B. W. Pruitt, G. M. Church, *Procedia Computer Science* **2016**, *80*, 1011, <https://doi.org/https://doi.org/10.1016/j.procs.2016.05.398>.

[6] Y. Erlich, D. Zielinski, *Science* **2017**, *355* (6328), 950, <https://doi.org/10.1126/science.aaj2038>.

[7] S. M. H. T. Yazdi, R. Gabrys, O. Milenkovic, *Scientific Reports* **2017**, *7* (1), 5011, <https://doi.org/10.1038/s41598-017-05188-1>.

[8] L. Organick, S. D. Ang, Y.-J. Chen, R. Lopez, S. Yekhanin, K. Makarychev, M. Z. Racz, G. Kamath, P. Gopalan, B. Nguyen, C. N. Takahashi, S. Newman, H.-Y. Parker, C. Rashtchian, K. Stewart, G. Gupta, R. Carlson, J. Mulligan, D. Carmean, G. Seelig, L. Ceze, K. Strauss, *Nature Biotechnology* **2018**, *36* (3), 242, <https://doi.org/10.1038/nbt.4079>.

[9] H. H. Lee, R. Kalhor, N. Goela, J. Bolot, G. M. Church, *Nature Communications* **2019**, *10* (1), 2383, <https://doi.org/10.1038/s41467-019-10258-1>.

[10] S. Chandak, J. Neu, K. Tatwawadi, J. Mardia, B. Lau, M. Kubit, R. Hulett, P. Griffin, M. Wootters, T. Weissman, H. Ji, in *ICASSP 2020 - 2020 IEEE International Conference on Acoustics, Speech and Signal Processing (ICASSP)* **2020**, 8822-8826.

[11] L. C. Meiser, P. L. Antkowiak, J. Koch, W. D. Chen, A. X. Kohll, W. J. Stark, R. Heckel, R. N. Grass, *Nature Protocols* **2020**, *15* (1), 86, <https://doi.org/10.1038/s41596-019-0244-5>.

[12] W. H. Press, J. A. Hawkins, S. K. Jones, J. M. Schaub, I. J. Finkelstein, *Proceedings of the National Academy of Sciences* **2020**, *117* (31), 18489, <https://doi.org/10.1073/pnas.2004821117>.

[13] W. Chen, M. A.-O. Han, J. Zhou, Q. Ge, P. Wang, X. Zhang, S. Zhu, L. Song, Y. A.-O. Yuan, (2053-714X (Electronic)).

[14] L. Ding, S. Wu, Z. Hou, A. Li, Y. Xu, H. Feng, W. Pan, J. Ruan, *National Science Review* **2023**, <https://doi.org/10.1093/nsr/nwad229>.

[15] L. Anavy, I. Vaknin, O. Atar, R. Amit, Z. Yakhini, *Nature Biotechnology* **2019**, *37* (10), 1229, <https://doi.org/10.1038/s41587-019-0240-x>.
